# Serum Oxidative Status in People with Obesity: Relation to Tissue Losses, Glucose Levels, and Weight Reduction

**DOI:** 10.3390/antiox12111923

**Published:** 2023-10-27

**Authors:** Beata Szlachta, Anna Birková, Tomasz Wielkoszyński, Alicja Gospodarczyk, Beáta Hubková, Maria Dydoń, Jolanta Zalejska-Fiolka

**Affiliations:** 1 Department of Biochemistry, Faculty of Medical Science, Zabrze Medical University of Silesia, 40-055 Katowice, Poland; b.szlachta@uk-brandenburg.de (B.S.); d201089@365.sum.edu.pl (A.G.); marysia.dydon@gmail.com (M.D.); jzalejskafiolka@sum.edu.pl (J.Z.-F.); 2 Department of Medical and Clinical Biochemistry, Pavol Jozef Šafárik University, 040 11 Košice, Slovakia; beata.hubkova@upjs.sk; 3 Wielkoszyński Medical Center, 41-300 Dąbrowa Górnicza, Poland; t.wielkoszynski@wielkoszynski.pl; 4 Doctoral School, Faculty of Medical Science, Zabrze Medical University of Silesia, 40-055 Katowice, Poland

**Keywords:** bioimpedance, weight loss, oxidative status, glycemia

## Abstract

Background: This work aims to study the effect of reductions in various body mass components on the oxidative, glycemic, and lipid parameters of people with obesity (PWO). Methods: A total of 53 PWO underwent a six-month individualized low-calorie diet combined with moderate exercise, during which anthropometric, biochemical, and oxidative parameters were measured. Probands were divided into groups based on weight, visceral fat area (VFA), total body water (TBW), and skeletal muscle mass (SMM) losses. Results: Weight reduction normalizes glycemia, but VFA reduction is less pronounced, while SMM and TBW reductions are more pronounced in patients with higher initial concentrations of glucose and fructosamine. Moreover, changes in oxidative parameters correlate with changes in glucose. Conclusions: Weight loss, regardless of the reduced tissue, decreases cardiovascular risk. We observed a significant change in almost all parameters related to the redox state. In general, parameters responsible for antioxidant action improved, and markers of oxidative damage decreased. Malondialdehyde, lipid peroxides, and total oxidative status levels can be considered biomarkers reflecting only the current severity of reactive oxygen species genesis processes. When considering the glycemic state, the results are not as clear due to the substantial differences between normoglycemic and hyperglycemic patients. Glycemic status is a factor playing a crucial role in weight reduction.

## 1. Introduction

At present, one of the world’s most serious health problems is obesity. According to the World Health Organization (WHO), obesity rates have almost tripled since 1975. In 2016, more than 1.9 billion adults were overweight, and of those, more than 650 million were obese. Globally, more than 39% of adults are overweight, and obesity occurs in 13% of them [1]. According to the Central Statistical Office, in Poland in 2019, more than 57% of adult people were overweight (overweight or obese), with excessive weight affecting men (65%) more than women (49%) [2]. Especially now, in the period after a nearly two-year pandemic and lockdown related to learning and remote work, this trend is more pronounced.

The prevalence of obesity is influenced by dietary and lifestyle changes in addition to genetic factors. The prevalence varies by region, but in developed countries, it affects the majority of the population. This fact confirms that environmental factors are the main factor influencing the development of obesity. Currently, an increasing trend in the prevalence of overweight and obesity can also be seen in developing countries due to the process of urbanization and the popularization of unhealthy lifestyles [3].

The WHO defines overweight as a body mass index (BMI) of 25.0 to 29.9 kg/m^2^, while obesity is defined as a BMI ≥ 30 kg/m^2^ [1]. However, BMI has its limitations, as it does not tell us about the distribution of body fat or lean body mass. It is well known that, unlike peripheral adipose tissue, excessive accumulation of visceral fat (so-called abdominal obesity) is a significant risk factor for cardiovascular diseases (CVDs). It contributes to the following complications: hypertension, insulin resistance, hyperinsulinemia, diabetes mellitus, dyslipidemia, and, eventually, atherosclerosis, ischemic heart disease, stroke, peripheral artery disease, and cancer, especially colorectal cancer. The mechanism linking obesity to other diseases has not yet been fully elucidated [4].

Obesity is a chronic disease with a complex etiology, associated with impaired lipid and glucose metabolism, reduced insulin sensitivity, abnormal inflammatory response, and low antioxidant capacity. Oxidative stress results from an imbalance between the action of pro-oxidant factors, or reactive oxygen species (ROS), and the antioxidants that counteract them. The main ROS include superoxide anion, hydroxyl radical, and hydrogen peroxide. Removal of free radicals is carried out by enzymatic and non-enzymatic antioxidants. Superoxide dismutase, catalase, peroxidase, glutathione transferase, and glutathione reductase are key antioxidant enzymes. In contrast, glutathione, vitamins A, C, and E, carotenoids, tocopherols, and tocotrienols are among the non-enzymatic antioxidants [5].

There is evidence that ROS stimulate adipogenesis, lipogenesis, and the differentiation of preadipocytes into mature adipocytes, leading to the deposition of white adipose tissue and the development of obesity. In addition, they are involved in the regulation of body weight by affecting hypothalamic neurons that control feelings of hunger and satiety [6,7]. In obesity, sources of oxidative stress include hyperglycemia, hyperleptinemia, chronic inflammation, low antioxidant activity, increased lipid levels, impaired mitochondrial function, endothelial dysfunction, and vitamin and mineral deficiencies [8]. Thus, it seems necessary to observe the oxidative status in obese patients before and after weight reduction in order to assess the effect of the applied therapy on reducing the level of harmful pro-oxidant factors.

A wide range of strategies are recommended to reduce the prevalence of obesity and thus prevent CVDs. Lifestyle modifications, changes in eating habits, and regular physical activity that will contribute to weight loss are extremely important. A diet low in saturated fatty acids (especially of animal origin), simple sugars, and salt and rich in fiber, raw vegetables, fruits, and polyunsaturated fatty acids is conducive to promoting health. In addition, many plant products contain natural antioxidant compounds such as polyphenols, flavonoids, isoflavones, carotenoids, capsaicinoids, isothiocyanates, and catechins. Therefore, a change in diet can help improve adipose tissue metabolism in obese people by reducing oxidative stress. The basis of effective therapy, in addition to an individualized diet, is the introduction of adequate physical activity, especially with the currently common sedentary lifestyle [9,10,11].

Due to the health consequences of obesity, weight reduction is recommended. However, attention should be paid to the patient’s health condition before starting weight reduction, and the process should be properly controlled. Weight reduction requires more than just weight control. It is known that losing adipose tissue brings benefits, but uncontrolled reduction can lead to body damage without improving its condition [9,12,13,14].

We hypothesized that glycemia and oxidative stress would normalize in PWO only after a reduction in the adipose tissue content. Therefore, this study aims to analyze the relationship between selected anthropometric and biochemical parameters and oxidative status in PWO undergoing weight loss intervention and, most importantly, compare the results obtained depending on what part of the body composition has become depleted during the reduction.

## 2. Materials and Methods

All procedures were approved by the Medical University of Silesia Ethics Committee in Katowice, Poland, No. KNW/0022/KB1/19/I/16. Written informed consent to participate in the study and publish the research results was obtained from all subjects. To avoid bias, for biochemical tests and statistical analysis, the samples were anonymized and numbered.

### 2.1. Study Population

**Figure 1:**
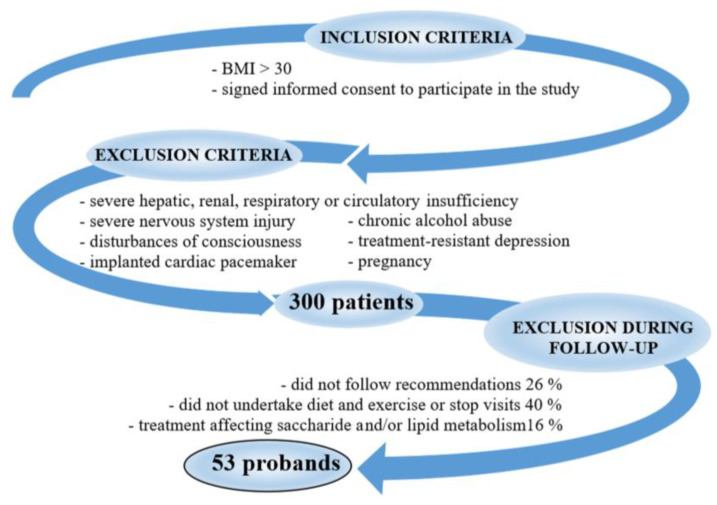
Study population. Legend: BMI—body mass index.

This study was a prospective, single-center study based on reports indicating that body mass reduction can ameliorate metabolic impairment. The study population, experimental groups, and inclusion/exclusion criteria are shown in Figure 1.

A total of 53 (37 women and 16 men) people with obesity volunteered and were qualified for the 6-month weight reduction program, which consisted of a balanced, individualized, low-calorie diet, moderate exercise, and health education.

### 2.2. Dietary and Physical Activity Intervention

Patients’ weight and waist/hip circumferences, as well as various other parameters, were measured during the six-month follow-up. They received motivation, support, and training on calorie calculation and food quality. Progress was monitored until a healthy body weight was reached or a 5–15% reduction in the initial weight was achieved. Initial data collection included a history of obesity, food preferences, health problems, and physical activity. Personalized instructions were given based on recommendations from the Polish National Food and Nutrition Institute, including an example of a seven-day menu, water and vegetable/fruit intake, and regular physical activity. The personalized diet consisted of balanced proportions of carbohydrates, total fat, and proteins. Patients were also advised to consume low- or medium-glycemic index snacks (vegetable or fruit) if needed.

### 2.3. Dividing Patients into Groups

No significant differences in glucose levels, BMI, VFA, or BFM were observed between male and female participants. Due to similar responses (Table S1), data from both sexes were analyzed collectively. Participants were divided into groups based on weight loss (WL), visceral fat area loss (VFA), total body water loss (TBW), and skeletal muscle mass loss (SMM) with cut-off lines for each category:
GroupSubgroups designationWeight lossWL < 10%WL > 10%Visceral fat area lossVFA < 15%VFA > 15%Total body water lossTBW < 5%TBW > 5%Skeletal muscle mass lossSMM < 5%SMM > 5%

Additionally, participants were divided into normo- (NG) and hyperglycemic (HG) groups based on the initial fasting glucose concentration.

### 2.4. Biochemical Assessment 

Following good laboratory practices, biochemical markers were assessed during the first and last visits. To avoid bias, all samples were anonymized and numbered. Fasting blood samples were collected at the Metabolic Clinic in Miasteczko Śląskie, Poland, from the cubital vein using clot activator tubes (2.7 mL) to obtain serum. After centrifugation (10 min, 3000 rpm, 4 °C), the serum was aliquoted and stored at −80 °C at the Department of Biochemistry Faculty of Medical Science Poland. Analytical methods underwent continuous intralaboratory quality control.

Glucose (Glc), fructosamine (FRU), total protein (PROT), t-CH, LDL-CH, HDL-CH, and TG levels were measured using a BS-200E biochemical analyzer (Mindray, Shenzhen, China) and Alpha Diagnostics reagents (San Antonio, TX, USA). Insulin was determined using INS-IRMA kits (KIP1251-KIP1254, DIA Source Immuno Assays S.A., Louvain, Belgium). The HOMA-IR coefficient was calculated as follows:

(fasting insulin concentration [µIU/mL] × fasting glucose concentration [mg/dL])/405.

### 2.5. Oxidative Status Parameters

Analysis of oxidative status parameters was performed in serum. All measurements were conducted using a PerkinElmer automated analyzer (PerkinElmer, Waltham, MA, USA). Superoxide dismutase (SOD) and its isoenzymes (MnSOD, CuZnSOD) were assessed using the Oyanagui method [15] and expressed in nitric units (NUs) per milliliter (mL). Total antioxidant capacity (TAC) was assessed using Erel’s protocol [16] and expressed in mmol/L. Ceruloplasmin (CER) concentration was assessed using the Richterich method [17] and expressed in mg/dL. Total oxidant status (TOS) was assessed using the Erel method [18] and expressed in µmol/L. Malondialdehyde (MDA) concentration was measured fluorometrically according to the methodology of Ohkawa, Ohishi, and Yagi [19] and expressed in µmol/L. Lipofuscin (LPS) concentration was determined according to the Jain methodology [20] and presented as relative units (relative fluorescence lipid extract, RF), where 100 RF corresponds to a fluorescence solution of 0.1 mg/L quinine sulfate in 0.05 M sulfuric acid. Protein sulfhydryl (PSH) groups were assessed using the method of Koster, Biemond, and Swaak [21] and expressed in μmol/L. Lipid peroxides (LPH) concentration was assessed using the method of Södergren et al. [22] and expressed in μmol/L.

### 2.6. Body Mass Parameters

Body mass parameters were measured using the InBody S10 body composition analyzer (Biospace, Cerritos, CA, USA), which utilizes bioelectrical impedance spectroscopy. This method was selected for its efficiency and accuracy [23,24]. The InBody S10 is certified to ISO 9001:2015, ISO 13485:2016, and medical certificates EN60601-1, EN60601-1-2, and CE MDD (Directive 93/42/EEC).

A standardized measurement protocol was implemented to obtain accurate bioimpedance results. This included recording age, sex, height, and body mass and conducting measurements at consistent time slots. Specific guidelines were followed, such as fasting for 3 h, abstaining from alcohol for 12 h, and avoiding medications that affect water balance. Subjects removed accessories, heavy clothing, and jewelry, and measurements were taken after standing upright for at least 5 min. Repeatability was assessed with 12 measurements on the same individual, resulting in coefficients of variation below 3% for all parameters.

### 2.7. Statistical Analysis

The results are presented before and after the 6-month weight reduction program. The calculated differences between these values are expressed as delta (Δ). All results are shown as the mean ± SD. Statistical analysis was performed with SPSS Statistics 22 (IBM, Armonk, NY, USA). The Kolmogorov–Smirnov test was used for normality testing. A paired *t*-test was used to determine differences in clinical parameters between values within groups before and after the dietary intervention. An unpaired *t*-test was used to determine differences in clinical parameters between groups, and the assumption of equal variances was tested using Levene’s Test for Equality of Variances. The Pearson correlation coefficient was used to express the strength and direction of linear relationships between two parameters. Statistical significance was assumed to be a *p*-value of <0.05.

## 3. Results

By comparing the obtained results before and after the study for the entire group of patients (Table 1), it was observed that only the levels of insulin, CRP, t-CH, LDL-CH, LPS, and CER did not change statistically significantly during the study. At the same time, an increase in HDL-CH, PSH, and TAC concentrations, as well as in SOD, MnSOD, and CuZnSOD activities, was observed. In contrast, the concentrations of glucose, FRU, insulin, TG, MDA, TOS, and LPH, the insulin resistance index HOMA-IR, and the new-AIP index were significantly reduced. In terms of body mass composition, a significant reduction was observed in all analyzed parameters (BMI, AC, VFA, PBF, TBW, and SMM) during the experiment.

**Figure 2:**
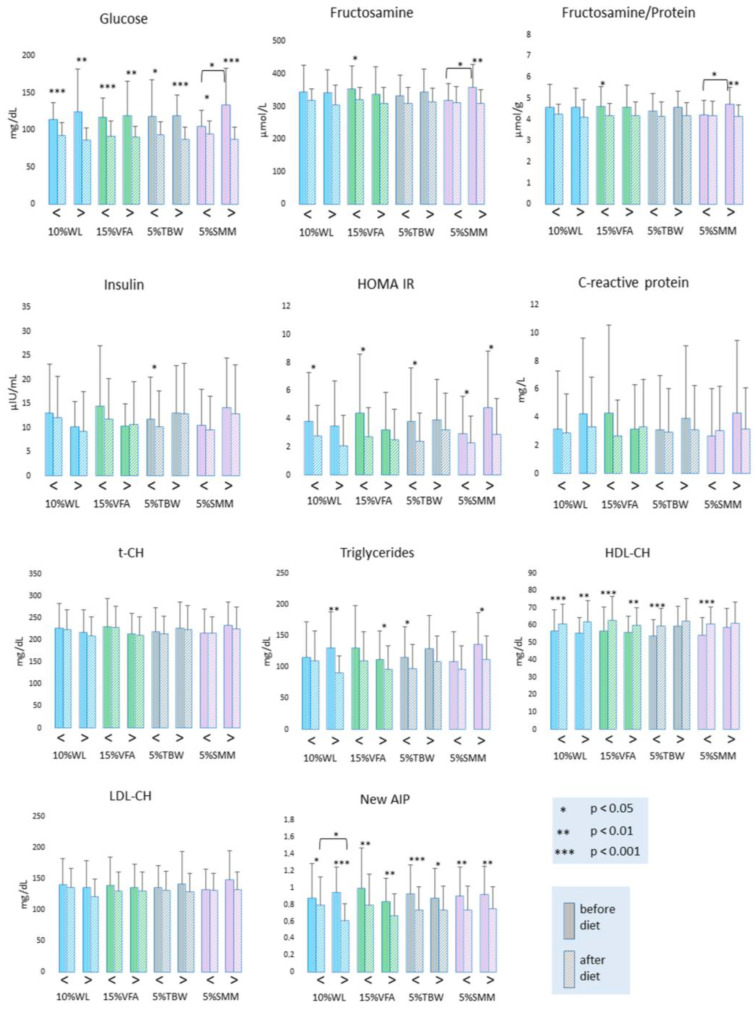
Parameters related to carbohydrate and lipid metabolism in obese patients enrolled in the study. Legend: HOMA-IR—Homeostatic Model Assessment for Insulin Resistance; t-CH—total cholesterol; HDL-CH—cholesterol in high-density lipoproteins; LDL-CH—cholesterol in low-density lipoproteins; AIP—atherogenic index of plasma.

Figure 2 and Table S2 present the results on saccharide and lipid metabolism parameters in various groups depending on the tissue compartment loss. At the beginning of the study, there was a significant difference in glucose and fructosamine levels only between patients in the SMM subgroups. Regardless of the subgrouping division used, a significant decrease in glucose levels was observed in all experimental groups. FRU levels decreased in the WL < 10%, VFA < 15%, TBW > 5%, and SMM > 5% subgroups. FRU/PROT levels decreased significantly only in the VFA < 15% and SMM > 5% subgroups. Before the body mass reduction program, there were no differences in HOMA-IR levels. After the study, HOMA-IR decreased in the WL < 10%, VFA < 15%, TBW < 5%, and SMM > 5% subgroups. HDL-CH significantly increased in all experimental groups except patients who lost more than 5% of TBW and SMM. A decrease in TG was observed in the WL > 10%, VFA > 15%, TBW < 5%, and SMM > 5% groups after the study. The new-AIP index decreased in all groups after the study.

### 3.1. Anthropometric Data of the Patients Included in the Study

The results on the selected body composition parameters in all study groups of PWO included in the study are presented in Table 2. Those who lost over 5% of SMM had significantly higher TBW levels before the study. A significant decrease in weight, BMI, WC, VFA, AC, and BFM was observed in all experimental groups without significant differences between subgroups. Statistically, significantly younger patients had greater TBW losses.

### 3.2. Oxidative Stress Parameter Levels Related to Specific Compartment Loss

**Figure 3:**
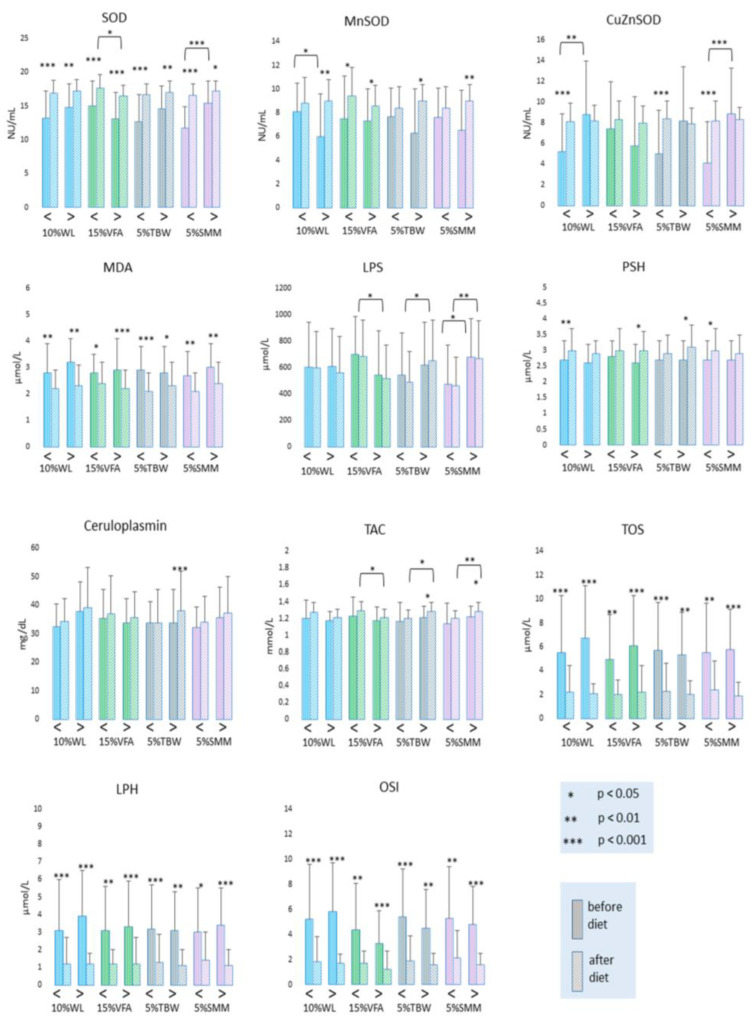
Antioxidant parameter levels related to specific compartment loss during the weight reduction program. Legend: SOD—superoxide dismutase; MnSOD—mitochondrial Mn-dependent superoxide dismutase; CuZnSOD—cytosolic Cu, Zn superoxide dismutase; MDA—malondialdehyde; LPS—lipofuscin; PSH—protein sulfhydryl groups; TAC—total non-enzymatic antioxidant capacity; TOS—total oxidant status; LPH—lipid peroxides; OSI—oxidative stress index.

Changes in oxidative stress parameters are demonstrated in Figure 3 and Table S3. Total serum superoxide dismutase (SOD) activity was significantly increased in each study group and subgroup. Before the start of the study, SOD activities were significantly lower (*p* < 0.001) in patients with less SMM loss compared to those with greater SMM loss. There was also a significant (*p* < 0.05) difference in SOD activities at the end of the study between those who reduced VFA by more than 15% and had lower SOD activities compared to patients with less VFA loss and higher SOD activities. A significant increase in the activity of the mitochondrial MnSOD isoenzyme was observed in the following groups: >10%WL (<0.01), <15%VFA and >15%VFA (*p* < 0.05), >5%TBW (*p* < 0.05) and >5% SMM (*p* < 0.01). Meanwhile, the activity of CuZnSOD increased in the opposite way in the WL<10% (*p* < 0.001), >15%VFA (*p* < 0.05), <5%TBW (<0.001), and <5%SMM (*p* < 0.001) groups. At the same time, a significant difference in MnSOD was observed before the study between those who were able to reduce their weight by more than 10% and those who lost less than 10% of their weight (*p* < 0.05). In addition, significantly higher CuZnSOD activities were observed before the study in the group that achieved a higher weight loss and a higher loss of skeletal muscle mass (*p* < 0.01 and <0.001, respectively). A significant increase in the CER concentration was observed only in the subgroup with >5%TBW loss (*p* < 0.05).

A significant decrease in the serum MDA concentration was observed in all experimental groups and subgroups, without differences between subgroups before and after the experiment.

Similarly, there were no significant changes in the LPS concentration in any subgroup when comparing the pre- and post-study values. However, significant changes occurred after the study (*p* < 0.05) between the <15%VFA subgroup with higher LPS values and the >15%VFA subgroup with a lower LPS concentration. There was also a significant difference between those who lost <5% and >5%TBW after the study (*p* < 0.05), while the LPS concentration was higher in patients with greater water loss. The LSP concentration also differed significantly depending on the loss of muscle mass, while the >5%SMM subgroup had significantly higher LPS concentrations at the beginning (*p* < 0.05) and at the end (*p* < 0.01) of the reduction program compared to the subgroup with a smaller loss of skeletal muscle mass. The concentration of PSH increased in <10%WL (*p* < 0.01), >15%VFA (*p* < 0.05), >5%TBW (*p* < 0.05), and <5%SMM (*p* < 0.001). The concentration of lipid peroxides (LPH) and TOS decreased significantly in all experimental groups and subgroups, without differences between subgroups before and at the end of the study. TAC increased significantly in the >5%TBW (*p* < 0.001) and >5%SMM groups (*p* < 0.05). There was a significant difference at the end of the study between the following subgroups: <15%VFA and >15%VFA (*p* < 0.05), <5%TBW and >5%TBW (*p* < 0.05), and <5%SMM and >5%SMM (*p* < 0.05). The concentrations of TAC were higher at the end of the study.

### 3.3. Correlation Analysis Revealed the Importance of Glucose Adjustment

For correlation analysis, results with significant changes in the above results from Table 1, Table 2, Tables S2 and S3 were chosen. The results are shown as delta (Table 3):

∆ *=* parameter before study (1) − parameter at the end of the study (2)

All results’ correlations showed no dependence of ∆VFA, ∆TBW, and ∆SMM on oxidative status parameters, glucose, and the chosen lipid parameters. There was a positive correlation between ∆weight and ∆TG (*p* < 0.05), ∆new-AIP index (*p* < 0.001), and ∆CuZnSOD (*p* < 0.05), and a negative correlation with ∆MnSOD (*p* < 0.05). There was also a positive correlation between ∆glucose and ∆SOD, ∆CuZnSOD, ∆MDA, and ∆LPH (*p* < 0.001, *p* = 0.008, *p* < 0.05, and *p* < 0.05, respectively).

**Figure 4:**
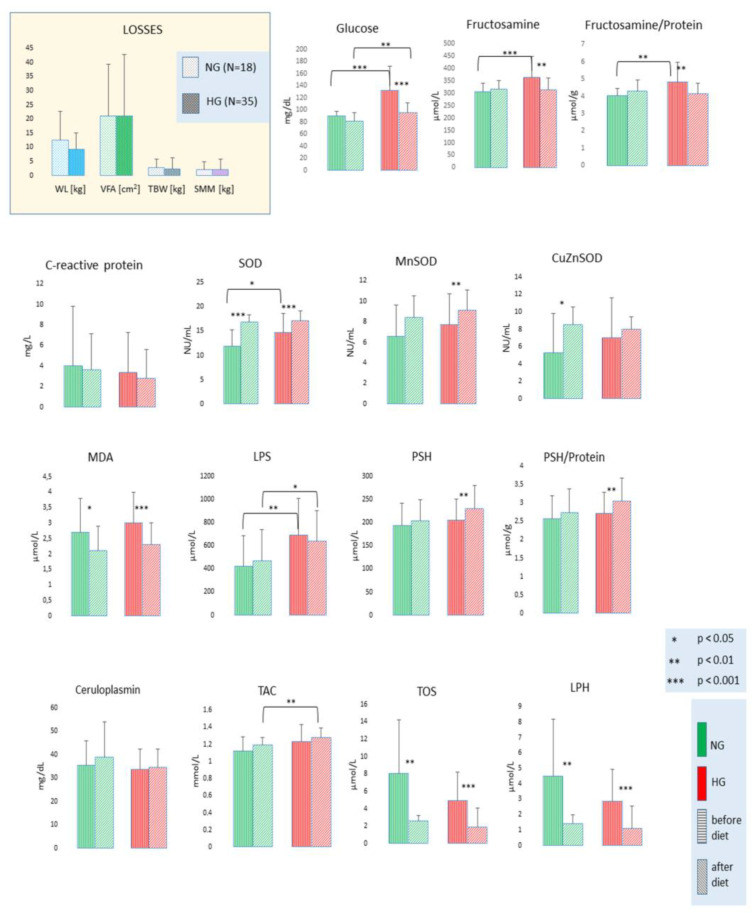
Changes in oxidative state—related parameters in normoglycemic and hyperglycemic patients during the weight reduction program. Legend: NG—normoglycemia patients; HG—hyperglycemia patients; WL—weight loss; VFA—visceral fat area; TBW—total body water; SMM—skeletal muscle mass; FRU—fructosamine; PROT—proteins; SOD—superoxide dismutase; MnSOD—mitochondrial Mn-dependent superoxide dismutase; CuZnSOD—cytosolic Cu, Zn superoxide dismutase; MDA—malondialdehyde; LPS—lipofuscin; PSH—protein sulfhydryl groups; CER—ceruloplasmin; TAC—total non-enzymatic antioxidant capacity; TOS—total oxidant status; LPH—lipid peroxides.

Considering the correlations between glucose and anti- and pro-oxidant parameters, we decided to show the changes in the oxidative status in the normo- and hyperglycemic groups (Figure 4, Table S4). Before the study, there were no differences in weight, VFA, TBW, and SMM loss between the normo- and hyperglycemic groups. In carbohydrate metabolism, significantly higher glucose and fructosamine levels were observed in the hyperglycemic group before the study. The concentration of those parameters decreased during the therapy, but the concentration of glucose in the hyperglycemic patients was still significantly higher than in the normoglycemic patients; however, those patients in the hyperglycemic group reached the reference value. The fructosamine-to-protein ratio was higher in the hyperglycemic group than in the normoglycemic group before the study and decreased during the study.

Total SOD activity was higher in the hyperglycemic group before the study; however, the SOD activity increased in both groups at the end of the study. In MnSOD and CuZnSOD activity, there were no differences between groups before the study. After the study, a significant increase was observed in MnSOD activity in the hyperglycemic group and in CuZnSOD activity in the normoglycemic group. MDA, TOS, and LPH concentrations decreased in both groups without observed differences between groups before the study. In LPS, higher values in the hyperglycemic group were observed before and after the study. The thiol group concentration (PSH and PSH/Protein) increased significantly only in the hyperglycemic group.

## 4. Discussion

The primary goal of weight loss is to reduce body weight, prevent weight regain, and achieve long-term maintenance of lower body weight. This goal is closely associated with health benefits in carbohydrate, lipid, and protein metabolism, as well as the body’s oxidative status [25]. In light of these considerations, our study aimed to investigate whether a weight reduction program could effectively modulate the oxidative status in PWO. Additionally, we aimed to determine whether the specific type of reduced tissue or body component would significantly impact serum oxidative status and carbohydrate and lipid profiles and how glycemia influences the study parameters.

Previous findings indicate that reducing visceral fat is the most favorable metabolically, whereas reducing skeletal muscle or water is less favorable [26,27,28]. Our previous research also revealed that weight reduction through a balanced diet and exercise is more effective in normoglycemic compared to hyperglycemic women with obesity. However, weight loss in hyperglycemic individuals led to an undesired loss of non-fat tissues, such as skeletal muscle mass, body protein content, and intracellular and total body water [27].

In this study, we found that regardless of the type of tissue reduced and the initial glucose levels, the glucose concentration decreased in all patient groups. However, those patients who lost more than 5% of SMM had the highest glucose and fructosamine levels and HOMA-IR before the program. This indicates the influence of glycemic status on tissue loss and supports our previous results [27].

It was shown that the type of tissue reduced did not affect t-CH and LDL-CH concentrations. However, at the same time, the HDL-CH concentration significantly increased in patients who reduced VFA and in those who reduced less than 5% of TBW and SMM. The lack of change in HDL-CH levels in patients who lost more than 5% of TBW and SMM suggests that significant loss of these tissues adversely affects cholesterol turnover. We also observed a significant reduction in TG levels in patients who lost more than 15% of VFA, which was an expected change. However, similar observations apply to patients who lost more than 5% SMM and less than 5% TBW. It is not known why such a change in TG levels was observed in those patients; hence, this requires further observations on a larger number of patients. In addition, a significant decrease in new-AIP was observed in all study groups, indicating that weight reduction reduces CVD risk regardless of the type of tissue reduced.

Analyzing body mass components of body weight, we observed a decrease in all analyzed parameters during the weight reduction in all patient groups, except for a decrease in total body water in patients who lost less than 5% of SMM. However, for these patients, the amount of total body water was lower before the weight reduction began. Moreover, patients who lost more than 5% of TBW during the program were significantly younger than those who lost less than 5% of TBW. Interestingly, those who lost more than 5% of SMM also experienced a significant reduction in TBW. This observation suggests a potential link between water loss, skeletal muscle mass loss, and glycemic status, which may be unfavorable.

An imbalance in the cell’s redox balance increases oxidative stress, resulting in the overproduction of reactive oxygen species (ROS). This frequently coexists with obesity, hyperglycemia, lipid disorders, and chronic inflammation. Not only obesity but also quick changes in body weight, whether through reduction or gain, can disrupt the delicate oxidative balance [5]. These alterations lead to increased production of free radical damage products, affecting many molecules and cellular components. The interaction between frequent hyperglycemia and oxidative stress can also trigger glycoxidation. Proteins, nucleic acids, and lipids are particularly susceptible to damage from ROS, with the hydroxyl radical primarily causing oxidative changes in proteins. In contrast, the superoxide anion radical and hydrogen peroxide contribute to other modifications, such as the oxidation of sulfhydryl groups. These reactions can generate nitrotyrosine and inhibit fibrinogen and tissue factor activity. Oxidative damage to thiol groups further disrupts cellular homeostasis, impacting the function of transmembrane proteins, enzymes, and transmitters [29].

Chronic oxidative stress triggers the expression of antioxidant enzymes in cells. Together with exogenous and endogenous antioxidants such as vitamins E, A, and C, bilirubin, and thiol groups, they help reduce the resulting imbalance [30].

As suggested by clinical experience, the greatest weight loss occurs in the first six to eight weeks, followed by slower progress. This is most likely related to patients’ stronger motivation at the beginning of weight reduction. Later, when the decrease in body mass is not so visible, motivation decreases, and to achieve the set goal, working with the patient seems crucial. Also, during this period, catabolic reactions and ROS formation are probably most pronounced. As weight stabilizes, these reactions diminish, and oxidation-reduction parameters normalize. Dietary restriction-induced weight loss reduces ROS generation by leukocytes and minimizes oxidative damage to lipids, proteins, and amino acids [31].

A well-known, major, and positive goal of weight loss is the reduction in visceral fat (VFA) and the resulting normalization of biochemical parameters. Our study reveals a significant increase in plasma sulfhydryl (PSH) concentrations in patients who lost over 15% of VFA, indicating an improved capacity to combat oxidative stress. Interestingly, patients with less SMM loss also exhibited an increased PSH concentration, suggesting a more effective reduction in oxidative stress in these patients as well.

Also, unexpectedly, regardless of the reduced tissue type, a decrease in other parameters reflecting ROS genesis processes, namely MDA, LPH, and TOS, was observed. At the same time, the concentration of lipofuscin, a late marker of cellular damage [32], did not follow similar changes. Moreover, hyperglycemic patients had significantly higher LPS concentrations before and after the study, suggesting its potential to predict the weight loss direction. Limited data explain the clinical relevance of LPS measurement in PWO, linking it to increased lipid peroxidation and aldehyde production, promoting cross-linking between proteins and phospholipids [33]. It seems highly probable that the LPS level could be an oxidative stress marker of obesity.

According to the glycemic state, the MDA concentration decreased in both the normo- and hyperglycemic groups after the weight reduction, indicating inhibition of lipid peroxidation regardless of glycemia. Simultaneously, the PSH concentration increased only in the hyperglycemic group, suggesting higher initial oxidative stress in PWO with hyperglycemia, effectively reduced by weight loss.

In addition to enzymatic antioxidants, a diet rich in fruits, vegetables, and oils provides exogenous antioxidants that improve the body’s antioxidant system, reducing the risk of metabolic disorders related to obesity [34]. TAC reflects the combined effect of various antioxidants in blood or tissue fluid [35]. Uric acid is one of the primary endogenous antioxidants determined using the above method. In our study, increased TAC values in patients losing more than 5% of TBW and SMM may be attributed to nucleic acid catabolism associated with muscle tissue loss. Moreover, a significantly higher TAC level was observed in hyperglycemic patients after the study. This can be considered an adverse effect of weight reduction. The literature on TAC levels shows conflicting results. Gać et al. found a negative correlation between BMI and TAC in hypertensive patients [36], while Jakubiak et al. observed a higher TAC in women with obesity with metabolic diseases, correlating with t-CH and TG levels [5]. TAC may serve as a marker for metabolic disorders in obesity, possibly related to hyperuricemia.

Superoxide dismutase (SOD) is a vital enzyme in the body’s defense against reactive oxygen species (ROS). Some researchers have proposed that when the body faces oxidative stress, it boosts SOD production as a protective measure to prevent oxidative damage to mitochondria [37].

In our study, we observed a significant increase in SOD activity across all subgroups undergoing weight reduction. However, the two isoenzymes, MnSOD and CuZnSOD, displayed different patterns. MnSOD activity increased in subgroups characterized by substantial weight loss and reductions in visceral fat (VFA), total body weight (TBW), and skeletal muscle mass (SMM). In contrast, CuZnSOD activity increased in subgroups with lower losses of these tissues. Notably, there was a negative correlation between changes in MnSOD activity and the amount of weight lost, while a positive correlation was observed between changes in CuZnSOD activity and weight. Patients who reduced more than 15% of VFA had lower SOD activity after the program, whereas those who lost more than 5% of SMM and 10% of their total body weight exhibited higher SOD and CuZnSOD activities before the program. Additionally, this study found that SOD activity was notably higher in individuals with hyperglycemia before starting the weight reduction program. This suggests that the reduction in skeletal muscle mass may lead to an increase in SOD activity due to the heightened production of superoxide radicals. These findings indicate that SOD activity could be a valuable marker for assessing oxidative status in individuals undergoing weight reduction. This aligns with previous research by Isogawa et al., who reported a negative correlation between SOD activity and body mass index (BMI). Lower SOD activity was associated with an increased carotid intima–media thickness, a well-known indicator of heightened cardiovascular risk. Interestingly, carotid plaque showed a positive correlation with SOD activity [38]. This relationship was further confirmed by Jakubiak et al., particularly in persons with a metabolically unhealthy phenotype, with the effect being slightly more pronounced in women than in men [5]. Yubero-Serrano et al. concluded that SOD activity could be the most relevant biomarker of oxidative stress in patients suffering from metabolic syndrome [39].

In our study, we also found something interesting. We noticed that patients who lost more than 5% of their total body water (TBW) had an increase in ceruloplasmin oxidase activity. This change was not connected to their blood sugar levels (glycemia) or any acute phase reactions in the body, as indicated by the hs-CRP levels, which did not show significant changes. Ceruloplasmin is a protein in the body with various roles, and its impact on the cardiovascular system and metabolic health is a topic of debate among researchers [40]. In our study, we did not find strong links between ceruloplasmin activity and other biochemical factors or changes in body tissues. This suggests that more research is necessary to better understand the significance of this finding and how it relates to the cardiovascular and metabolic aspects of health.

## 5. Conclusions

1. The observed outcomes are a consequence of various processes that exacerbate and inhibit the severity of oxidative stress. On the one hand, obesity, hyperlipidemia, inflammation, and the process of active weight reduction generate increased amounts of reactive oxygen species (ROS); on the other hand, changes in dietary habits involving the introduction of a balanced diet based on healthy food items (vegetables, fruits, fish, oils, nuts, and seeds), reduced caloric intake, and moderate physical activity have led to an improvement in redox balance parameters;
2. Regarding tissue loss, the levels of total cholesterol (t-CH) and low-density lipoprotein cholesterol (LDL-CH) did not change significantly. An interesting observation is that greater muscle losses were associated with a significant decrease in triglyceride (TG) levels, while smaller muscle losses were linked to a significant increase in high-density lipoprotein cholesterol (HDL-CH);
3. A different pattern of changes in superoxide dismutase (SOD) isoenzymes’ activity was observed. Lower mitochondrial isoenzyme activity at the outset was associated with greater losses of skeletal muscle and water, in contrast to cytosolic isoenzyme activity, which was significantly lower before the diet in those who lost less water and skeletal muscle during the reduction;
4. Glucose levels play a significant role in determining the course of weight reduction and influencing the redox state. Hyperglycemia is associated with higher total SOD levels, and improvements in glycemia lead to a significant increase in mitochondrial SOD isoenzyme activity;
5. Overall, weight reduction is beneficial and leads to a decreased risk of cardiovascular diseases, but the pool of active antioxidant molecules is higher in cases of hyperglycemia, and it increases even more after glycemia improvement and weight reduction;
6. It also appears from this study that body weight alone is not an adequate indicator to predict the glycemic and redox statuses of patients.

### Study Limitation

Despite the admission of 300 eligible patients to the program, only 18% (53 men and women) were included in the study and successfully completed the therapy. The rest of the patients were excluded: 16% took medicine affecting carbohydrate and/or lipid metabolism; 26% did not follow the recommendations; 40% did not follow the recommended diet and exercise (after the first visit, they were maximum twice and did not appear anymore). In addition, we evaluated only the available data, i.e., from the beginning and the end of the study.

## Figures and Tables

**Table 1:** General description of the whole group of probands. Data are expressed as the mean ± SD. A *t*-test was used for the comparison of parameters before and after the diet. The difference is significant if *p* < 0.05.

	Before Diet	After Diet	*p*
Weight (kg)	101.0 ± 21.0	90.7 ± 19.0	<0.001
BMI (kg/m^2^)	36.1 ± 6.5	32.5 ± 6.1	<0.001
AC (cm)	40.5 ± 6.2	35.7 ± 3.9	<0.001
WC (cm)	109.8 ± 14.6	98.9 ± 14.5	<0.001
VFA (cm^2^)	142.2 ± 34.3	115.5 ± 33.5	<0.001
PBF (%)	39.9 ± 9.1	36.6 ± 9.5	<0.001
TBW (L)	44.2 ± 9.5	41.2 ± 8.7	<0.001
SMM (kg)	33.8 ± 7.7	31.2 ± 6.9	<0.001
Glucose (mg/dL)	117.7 ± 38.7	90.6 ± 16.9	<0.001
FRU (umol/L)	344.8 ± 76.8	314.6 ± 44.6	<0.01
FRU/PROT	4.58 ± 1.03	4.2 ± 0.62	<0.05
INS (µIU/mL)	12.0 ± 8.8	11.0 ± 8.5	ns
HOMA-IR	3.70 ± 3.38	2.53 ± 2.16	<0.01
CRP (mg/L)	3.58 ± 4.6	3.08 ± 3.02	ns
t-CH (mg/dL)	223.1 ± 54.6	218.0 ± 44.9	ns
TG (mg/dL)	121.3 ± 56.8	102.9 ± 41.8	<0.01
HDL-CH (mg/dL)	56.3 ± 11.0	61.2 ± 11.6	<0.001
LDL-CH (mg/dL)	138.8 ± 41.6	129.9 ± 30.0	ns
New-AIP	0.90 ± 0.37	0.73 ± 0.31	<0.001
SOD (NU/mL)	13.8 ± 3.9	17.0 ± 1.8	<0.001
MnSOD (NU/mL)	7.4 ± 3.0	8.9 ± 2.0	<0.01
CuZnSOD (NU/mL)	6.4 ± 4.6	8.2 ± 1.7	0.011
CER (mg/dL)	34.4 ± 9.1	36.1 ± 10.7	ns
TAC (mmol/L)	1.19 ± 0.19	1.25 ± 0.11	<0.05
TOS (μmol/L)	5.94 ± 4.65	2.13 ± 1.86	<0.001
OSI (TOS/TAC)	5.42 ± 4.19	1.80 ± 1.65	<0.001
MDA (μmol/L)	2.93 ± 1.06	2.24 ± 0.74	<0.001
LPH (μmol/L)	3.39 ± 2.79	1.19 ± 1.27	<0.001
LPS (RF)	604.2 ± 320.9	582.8 ± 273.4	ns
PSH (μmol/L)	202.0 ± 45.7	221.3 ± 49.4	<0.01
PSH/g of protein (μmol/g)	2.67 ± 0.60	2.94 ± 0.64	<0.01

Legend: X_1—before program; X_2—after program; ns—not significant; BMI—body mass index; AC—arm circumference; WC—waist circumference; VFA—visceral fat area; PBF—body fat percentage; TBW—total body water; SMM—skeletal muscle mass; FRU—fructosamine; PROT—proteins; INS—insulin; HOMA-IR—Homeostatic Model Assessment for Insulin Resistance; CRP—C-reactive protein; t-CH—total cholesterol; TG—triacylglycerols; HDL-CH—cholesterol in high-density lipoproteins; LDL-CH—cholesterol in low-density lipoproteins; AIP—atherogenic index of plasma; SOD—superoxide dismutase; MnSOD—mitochondrial Mn-dependent superoxide dismutase; CuZnSOD—cytosolic Cu, Zn superoxide dismutase; MDA—malondialdehyde; LPS—lipofuscin; PSH—protein sulfhydryl groups; CER—ceruloplasmin; TAC—total non-enzymatic antioxidant capacity; TOS—total oxidant status; LPH—lipid peroxides; OSI—oxidative stress index.

**Table 2:** Selected body composition parameters in individual groups of obese patients. Statistical analysis of age and anthropometrical parameters.

		Weight Loss		VFA Loss		TBW Loss		SMM Loss	
		WL < 10%	WL > 10%	VFA < 15%	VFA > 15%	TBW < 5%	TBW > 5%	SMM < 5%	SMM > 5%
Parameter	X	N = 34	N = 19	N = 21	N = 31	N = 27	N = 20	N = 24	N = 24
Age (y)		49 ± 12	46 ± 10 ^# ns^	50 ± 11	46 ± 11 ^# ns^	50 ± 11	44 ± 12 ^# < 0.05^	50 ± 11	47 ± 9 ^# ns^
Height (cm)		166 ± 8	168 ± 9 ^# ns^	168 ± 9	167 ± 8 ^# ns^	165 ± 7	168 ± 9 ^# ns^	165 ± 7	168 ± 8 ^# ns^
Weight (kg) *p* (1 vs. 2)	1	99.3 ± 22.1	104.1 ± 18.9 ^# ns^	102.7 ± 21.6	100.1 ± 21.2 ^# ns^	98.9 ± 19.8	105.5 ± 24.1 ^# ns^	97.7 ± 20.3	104.8 ± 21.8 ^# ns^
	2	93.0 ± 20.6	86.7 ± 15.4 ^# ns^	95.1 ± 19.8	87.7 ± 18.4 ^# ns^	89.1 ± 19.4	93.0 ± 19.9 ^# ns^	89.0 ± 19.6	92.5 ± 19.2 ^# ns^
		<0.001	<0.001	<0.001	<0.001	<0.001	<0.001	<0.001	<0.001
BMI (kg/m^2^) *p* (1 vs. 2)	1	35.8 ± 7.2	36.7 ± 5.1 ^# ns^	36.3 ± 6.4	36.0 ± 6.7 ^# ns^	36.2 ± 6.8	37.1 ± 6.6 ^# ns^	36.1 ± 6.8	36.9 ± 6.3 ^# ns^
	2	33.6 ± 6.7	30.6 ± 4.1 ^# < 0.05^	33.7 ± 6.2	31.6 ± 6.0 ^# ns^	32.6 ± 6.8	32.8 ± 5.8 ^# ns^	32.9 ± 6.7	32.6 ± 5.8 ^# ns^
		<0.001	<0.001	<0.001	<0.001	<0.001	<0.001	<0.001	<0.001
WC (cm) *p* (1 vs. 2)	1	109.2 ± 15.4	110.8 ± 13.4 ^# ns^	112.1 ± 15.6	118.3 ± 14.1 ^# ns^	109.4 ± 15.6	111.0 ± 15.0 ^# ns^	107.5 ± 15.9	112.8 ± 14.1 ^# ns^
	2	101.6 ± 14.0	94.0 ± 14.4 ^# ns^	102.1 ± 15.2	96.5 ± 14.0 ^# ns^	99.2 ± 15.5	98.6 ± 14.9 ^# ns^	97.6 ± 14.8	100.2 ± 15.0 ^# ns^
		<0.001	<0.001	<0.001	<0.001	<0.001	<0.001	<0.001	<0.001
TBW (L) *p* (1 vs. 2)	1	43.5 ± 9.8	45.4 ± 9.1 ^# ns^	45.6 ± 10.2	43.5 ± 9.1 ^# ns^	41.3 ± 6.7	47.5 ± 11.1 ^# ns^	40.5 ± 6.9	47.5 ± 9.9 ^# < 0.001^
	2	39.9 ± 8.5	42.9 ± 8.9 ^# ns^	43.3 ± 9.4	40.0 ± 8.2 ^# ns^	40.3 ± 7.0	42.4 ± 10.9 ^# ns^	39.6 ± 7.4	42.5 ± 9.8 ^# ns^
		<0.001	<0.05	0.001	<0.01	ns	<0.001	ns	<0.001
VFA (cm^2^) *p* (1 vs. 2)	1	138.5 ± 33.7	148.8 ± 35.3 ^# ns^	141.3 ± 35.1	142.2 ± 34.8 ^# ns^	143.3 ± 32.9	144.8 ± 36.8 ^# ns^	142.5 ± 32.5	115.59 ± 34.0 ^# ns^
	2	117.7 ± 33.3	111.6 ± 34.4 ^# ns^	130.1 ± 29.4	105.6 ± 32.9 ^# <0.001^	115.9 ± 35.1	115.4 ± 32.6 ^# ns^	115.6 ± 34.0	116.2 ± 33.4 ^# ns^
		<0.001	<0.001	<0.001	<0.001	<0.001	<0.001	<0.001	<0.001
AC (cm) *p* (1 vs. 2)	1	40.2 ± 7.1	40.8 ± 4.4 ^# ns^	40.3 ± 4.9	40.7 ± 7.2 ^# ns^	39.9 ± 6.4	42.1 ± 6.6 ^# ns^	39.5 ± 6.5	41.8 ± 6.2 ^# ns^
	2	36.4 ± 4.4	34.7 ± 3.0 ^# ns^	38.2 ± 4.0	34.3 ± 3.1 ^# < 0.001^	35.5 ± 4.4	36.0 ± 3.2 ^# ns^	35.4 ± 4.2	36.3 ± 3.3 ^# ns^
		<0.001	<0.001	<0.001	<0.001	<0.001	<0.001	<0.001	<0.001
BFM (kg) *p* (1 vs. 2)	1	40.1 ± 15.5	42.4 ± 13.2 ^# ns^	40.6 ± 13.6	40.8 ± 15.7 ^# ns^	42.8 ± 14.9	40.7 ± 15.0 ^# ns^	42.7 ± 15.0	40.0 ± 13.5 ^# ns^
	2	36.8 ± 12.1	28.9 ± 9.7 ^# < 0.01^	39.5 ± 12.1	29.9 ± 10.1 ^# < 0.001^	32.6 ± 13.9	34.6 ± 7.6 ^# ns^	33.3 ± 16.6	33.7 ± 9.3 ^# ns^
		<0.01	<0.001	<0.001	<0.001	<0.001	<0.05	<0.001	<0.01

Legend: X_1—before program; X_2—after program; ns—not significant; BMI—body mass index; AC—arm circumference; WC—waist circumference; VFA—visceral fat area; BFM—body fat mass; TBW—total body water; SMM—skeletal muscle mass. Data are expressed as the mean ± SD. For comparisons of differences between measurements before the diet and after the diet within groups (*p*(1 vs. 2)) and between groups (# *p*), a *t*-test was used.

**Table 3:** The correlation between selected body mass parameters and lipids, glucose level, and oxidative status parameters. Pearson’s coefficient r expresses the strength of the correlation. The correlation is significant if *p* < 0.05.

Biochemical Parameters	Pearson’s r Significance *p*	ΔWeight	ΔVFA	ΔTBW	ΔSMM	ΔGlc	ΔCH	ΔTG
ΔGlucose	r	0.207	0.028	0.214	0.166		0.177	0.141
	p	ns	ns	ns	ns		ns	ns
Δt-CH	r	0.094	−0.011	−0.014	0.022	0.177		
	p	ns	ns	ns	ns	ns		
ΔTG	r	0.284	0.087	−0.017	0.056	0.141	0.558	
	p	<0.05	ns	ns	ns	ns	<0.0001	
ΔLDL-CH	r	0.107	0.077	−0.131	−0.128	0.188	0.949	0.339
	p	ns	ns	ns	ns	ns	<0.0001	<0.05
ΔHDL-CH	r	−0.062	0.032	0.111	0.129	0.051	0.359	0.055
	p	ns	ns	ns	ns	ns	<0.05	ns
ΔNew-AIP	r	0.449	0.12	−0.115	−0.054	0.07	0.472	0.783
	p	<0.01	ns	ns	ns	0 ns	<0.01	<0.0001
ΔSOD	r	0.174	0.012	0.113	0.104	0.475	0.16	0.136
	p	ns	ns	ns	ns	<0.0001	ns	ns
ΔMnSOD	r	−0.305	0.104	−0.151	−0.153	−0.039	0.038	−0.101
	p	<0.05	ns	ns	ns	ns	ns	ns
ΔCuZnSOD	r	0.346	−0.068	0.19	0.186	0.368	0.087	0.171
	p	<0.05	ns	ns	ns	<0.01	ns	ns
ΔMDA	r	0.165	0.144	−0.152	−0.142	0.349	0.037	0.007
	p	ns	ns	ns	ns	<0.05	ns	ns
ΔPSH/PROT	r	0.06	0.012	0.014	0.008	−0.066	0.107	0.08
	p	ns	ns	ns	ns	ns	ns	ns
ΔTOS	r	0.166	0.093	0.197	0.167	0.214	−0.120	0.03
	p	ns	ns	ns	ns	ns	ns	ns
ΔLPH	r	0.175	0.058	0.199	0.17	0.279	−0.097	0.019
	p	ns	ns	ns	ns	<0.05	ns	ns

Legend: ns—not significant; PROT—proteins; t-CH—total cholesterol; TG—triacylglycerols; HDL-CH—cholesterol in high-density lipoproteins; LDL-CH—cholesterol in low-density lipoproteins; AIP—atherogenic index of plasma; SOD—superoxide dismutase; MnSOD—mitochondrial Mn-dependent superoxide dismutase; CuZnSOD—cytosolic Cu, Zn superoxide dismutase; MDA—malondialdehyde; PSH—protein sulfhydryl groups; TOS—total oxidant status; LPH—lipid peroxides.

## Data Availability

The database of aggregated statistics ready for analysis is stored in a secure, confidential, and password-protected repository on the server of the Medical University of Silesia. The data were anonymized. Completely non-identifiable records may be made available to interested persons/organizations upon request at jzalejskafiolka@sum.edu.pl.

## Supplementary Materials

The following supporting information can be downloaded at https://www.mdpi.com/article/10.3390/antiox12111923/s1, Table S1: Gender analysis of particular compartments loss. Statistical analysis of gender dependent compartment loss. Data are expressed as the mean ± SD, for comparison differences between measurements before the diet and after the diet within groups (*p* (1 vs. 2)) and between groups (#*p*), a *t*-test was used; Table S2: Statistical analysis of parameters related to saccharide and lipid metabolism in individual groups of obese patients. Data are expressed as the mean ± SD, for comparison differences between measurements before the diet and after the diet within groups (*p*(1 vs. 2)) and between groups (#*p*), a *t*-test was used; Table S3: Statistical analysis of selected anti- and pro-oxidative parameters in individual groups of obese patients. Data are expressed as the mean ± SD, for comparison differences between measurements before the diet and after the diet within groups (*p*(1 vs. 2)) and between groups (#*p*), a *t*-test was used; Table S4: The differences between chosen parameters in normo- and hyperglycemia groups. Data are expressed as the mean ± SD, for comparison differences between measurements before the diet and after the diet within groups (*p* (1 vs. 2)) and between groups (#*p*), a *t*-test was used.
