# Supplementary material for: Serum Oxidative Status in People with Obesity: Relation to Tissue Losses, Glucose Levels, and Weight Reduction
Source: Antioxidants (Basel). 2023 Oct 27;12(11):1923. doi: 10.3390/antiox12111923 (PMC10669655; doi:10.3390/antiox12111923)
Supplement: Supplementary file 1 [file antioxidants-12-01923-s001.zip › antioxidants-2670485-supplementary.pdf]

Supplementary Materials

Table S1. Gender analysis of particular compartments loss. Statistical analysis of gender dependent compartment loss. Data are expressed as the mean ± SD, for comparison differences between measurements before the diet and after the diet within groups (*p*(1vs2)) and between groups (#*p*), a *t*-test was used. .

Parameter	X	Men (*n* = 16)	Women (*n* = 37)	#*p*
Height (cm)		176.3±6.0	163.5±5.9	**<0.001**
Weight	1	113.2±23.0	95.4±18.0[3]	**<0.01**
kg	2	103.8±20.6	84.5±14.6	**<0.001**
p (1vs2)		**<0.001**	**<0.001**	
Δ weight (%)		8.2±3.7	10.9±6.9	ns
BMI	1	36.3±6.4	35.7±6.2	ns
kg/m²	2	33.3±6.0	31.6±5.4	ns
p (1vs2)		**<0.001**	**<0.001**	
Δ BMI (%)		8.2±3.7	10.9 ±6.9	ns
VFA	1	149.8±41.1	138.7±31.6	ns
cm²	2	126.7±40.4	110.0±29.6	ns
p (1vs2)		**<0.001**	**<0.001**	
Δ VFA (%)		16.1±12.1	20.1±11.9	ns
SMM	1	42.0±7.7	30.8±5.1	**<0.001**
kg	2	39.9±6.8	28.2±3.7	**<0.001**
p (1vs2)		**<0.05**	**<0.001**	
Δ SMM (%)		4.7±5.1	7.8±8.7	ns
TBW	1	54.8±9.2	40.0±6.0	**<0.001**
kg	2	51.1±8.4	37.4±4.8	**<0.001**
p (1vs2)		**<0.01**	**<0.001**	
Δ TBW (%)		4.0±4.7	7.0±7.3	ns

Legend: X_1 – before program; X_2 – after program; ns – not significant; BMI – body mass index; VFA – Visceral fat area; SMM – Skeletal muscle mass; TBW – Total body water.

Table S2. Statistical analysis of parameters related to saccharide and lipid metabolism in individual groups of obese patients. Data are expressed as the mean ± SD, for comparison differences between measurements before the diet and after the diet within groups (p(1vs2)) and between groups (#p), a *t*-test was used. .

		WEIGHT LOSS		VISCERAL FAT AREA LOSS		TOTAL BODY WATER LOSS		SKELETAL MUSCLE MASS LOSS	
		**WL<10%** *n* = 34	**WL>10% #p** *n* = 19	**VFA<15%** *n* = 21	**VFA>15% #*p*** *n* = 31	**TBW<5%** *n* = 27	**TBW>5% #*p*** *n* = 20	**SMM<5%** *n* = 24	**SMM>5% #p** *n* = 24
HOMA IR	1	3.82±3.49	3.48±3.24$^{\#\ ns}$	4.4±4.2	3.2±2.7$^{\#\ ns}$	3.80±3.81	3.90±3.21$^{\#\ ns}$	2.93±2.68	4.81±4.0$^{\#\ ns}$
	2	2.80±2.13	2.07±2.18$^{\#\ ns}$	2.7±2.1	2.5±2.2$^{\#\ ns}$	2.41±1.99	2.88±2.62$^{\#\ ns}$	2.30±1.89	2.88±2.55$^{\#\ ns}$
p (1vs2)		**<0.05**	ns	**<0.05**	ns	**<0.05**	ns	**<0.05**	**<0.05**
Glucose	1	113.6±22.9	124.9±57.4$^{\#\ ns}$	117.3±25.5	118.8±46.3$^{\#\ ns}$	118.2±49.0	119.6±27.1$^{\#\ ns}$	105.3±21.3	133.9±48.7$^{\#<0.05}$
[mg/dl]	2	92.9±17.3	86.6±15.9$^{\#\ ns}$	91.1±21.0	90.5±14.3$^{\#\ ns}$	93.3±17.9	87.4±16.5$^{\#\ ns}$	94.4±17.6	87.3±16.1$^{\#\ ns}$
p (1vs2)		**<0.001**	**<0.01**	**<0.001**	**<0.01**	**<0.05**	**<0.001**	**<0.05**	**<0.001**
FRU	1	345.7±81.4	343.2±69.8$^{\#\ ns}$	354.9±69.4	338.5±83.0$^{\#\ ns}$	333.9±63.0	344.3±70.5$^{\#\ ns}$	319.0±52.3	358.8±71.2$^{\#<0.05}$
[umol/l]	2	319.6±33.5	305.8±59.2$^{\#\ ns}$	321.2±38.1	310.2±49.4$^{\#\ ns}$	309.9±47.7	314.1±43.3$^{\#\ ns}$	312.4±49.1	310.5±41.4$^{\#\ ns}$
p (1vs2)		**<0.05**	ns	**<0.05**	ns	ns	**<0.05**	ns	**<0.01**
FRU/PROT	1	4.57±1.11	4.58±0.90$^{\#\ ns}$	4.63±0.93	4.57±1.04$^{\#\ ns}$	4.40±0.82	4.57±0.75$^{\#\ ns}$	4.23±0.69	4.74±0.78$^{\#<0.05}$
	2	4.24±0.48	4.12±0.83$^{\#\ ns}$	4.19±0.58	4.17±0.65$^{\#\ ns}$	4.15±0.70	4.19±0.59$^{\#\ ns}$	4.17±0.69	4.15±0.53$^{\#\ ns}$
p (1vs2)		ns	ns	**<0.05**	ns	ns	ns	ns	**<0.01**

INS	1	13.0±10.2	10.25±5.1$^{\# ns}$	14.5±12.5	10.3±4.7$^{\# ns}$	11.8±8.7	13.1±9.8$^{\# ns}$	10.5±7.4	14.2±10.3$^{\# ns}$
[μIU/ml]	2	12.1±8.6	9.2±8.3$^{\# ns}$	11.7±8.4	10.6±8.9$^{\# ns}$	10.2±7.5	12.9±10.4$^{\# ns}$	9.5±7.1	12.9±10.1$^{\# ns}$
p (1vs2)		ns	ns	ns	ns	**<0.05**	ns	ns	ns
CRP	1	3.19±4.13	4.27±5.39$^{\# ns}$	4.33±6.24	3.15±3.17$^{\# ns}$	3.10±3.87	3.92±5.18$^{\# ns}$	2.66±3.37	4.31±5.17$^{\# ns}$
[mg/l]	2	2.92±2.73	3.34±3.53$^{\# ns}$	2.69±2.56	3.32±3.37$^{\# ns}$	2.96±3.09	3.14±3.15$^{\# ns}$	3.05±3.17	3.15±2.96$^{\# ns}$
p (1vs2)		ns	ns	ns	ns	0.86	ns	ns	ns
t-CH	1	226.3±57.3	217.6±50.59$^{\# ns}$	230.7±63.3	214.7±45.3$^{\# ns}$	219.6±53.4	226.9±60.2$^{\# ns}$	216.3±53.2	232.6±53.1$^{\# ns}$
[mg/dl]	2	223.1±45.5	209.5±43.7$^{\# ns}$	227.8±48.9	211.2±41.4$^{\# ns}$	214.4±40.2	224.4±53.7$^{\# ns}$	215.1±37.2	225.8±49.3$^{\# ns}$
P (1vs2)		ns	ns	ns	ns	ns	ns	ns	ns
TG	1	115.9±56.5	130.7±57.5$^{\# ns}$	130.5±67.8	112.1±45.7$^{\# ns}$	115.4±48.6	129.4±52.9$^{\# ns}$	109.3±47.5	135.8±50.6$^{\# ns}$
[mg/dl]	2	110.0±47.3	90.6±27.1$^{\# ns}$	110.1±46.0	96.1±37.5$^{\# ns}$	97.9±38.3	109.1±40.4$^{\# ns}$	96.1±37.4	111.6±38.3$^{\# ns}$
p (1vs2)		ns	**<0.01**	ns	**<0.05**	**<0.05**	ns	ns	**<0.05**
HDL - CH	1	56.8±12.3	55.6±8.7$^{\# ns}$	56.8±13.8	56.0±9.2$^{\# ns}$	53.7±9.6	59.4±11.5$^{\# ns}$	54.1±10.2	58.6±11.0$^{\# ns}$
[mg/dl]	2	60.8±11.5	61.9±12.2$^{\# ns}$	62.9±13.8	60.1±9.9$^{\# ns}$	59.6±10.0	62.3±13.0$^{\# ns}$	60.7±10.0	61.2±12.4$^{\# ns}$
p (1vs2)		**<0.001**	**<0.01**	**<0.001**	**<0.01**	**<0.001**	ns	**<0.001**	ns
LDL - CH	1	140.6±41.3	135.9±42.9$^{\# ns}$	139.4±45.4	135.9±37.6$^{\# ns}$	136.0±34.7	141.6±51.4$^{\# ns}$	132.6±32.7	147.9±46.1$^{\# ns}$
[mg/dl]	2	136.0±29.9	120.6±28.6$^{\# ns}$	130.1±30.5	129.8±30.4$^{\# ns}$	130.9±31.3	128.7±29.2$^{\# ns}$	130.6±27.5	132.8±28.2$^{\# ns}$
p (1vs2)		ns	ns	ns	ns	ns	ns	ns	ns
New AIP	1	0.88±0.41	0.94±0.30$^{\# ns}$	0.99±0.48	0.83±0.28$^{\# ns}$	0.93±0.34	0.88±0.35$^{\# ns}$	0.9±0.34	0.92±0.33$^{\# ns}$
	2	0.79±0.34	0.61±0.20$^{\#<0.05}$	0.79±0.37	0.67±0.26$^{\# ns}$	0.73±0.28	0.73±0.29$^{\# ns}$	0.73±0.29	0.75±0.26$^{\# ns}$
p (1vs2)		**<0.05**	**<0.001**	**<0.01**	**<0.01**	**<0.001**	**<0.05**	**<0.01**	**<0.01**

Legend: X_1 – before program; X_2 – after program; ns – not significant; HOMA-IR – Homeostatic Model Assessment for Insulin Resistance; FRU – fructosamine; PROT – proteins; INS – insulin; CRP – C-reactive protein; t-CH – total cholesterol; TG – triacylglycerols; HDL-CH – cholesterol in high density lipoproteins; LDL-CH - cholesterol in low density lipoproteins; AIP – atherogenic index of plasma.

Table S3. Statistical analysis of selected anti- and pro-oxidative parameters in individual groups of obese patients. Data are expressed as the mean ± SD, for comparison differences between measurements before the diet and after the diet within groups (p(1vs2)) and between groups (#p), a *t*-test was used. .

PARAMETER	X	WEIGHT LOSS		VISCERAL FAT AREA LOSS		TOTAL BODY WATER LOSS		SKELETAL MUSCE MASS LOSS	
		**WL<10%** *n* **= 34**	**WL>10% #p** *n* **= 19**	**VFA<15%** *n* **= 21**	**VFA>15% #p** *n* **= 31**	**TBW<5%** *n* **= 27**	**TBW>5% #p** *n* **= 20**	**SMM<5%** *n* **= 24**	**SMM>5% #p** *n* **= 24**
SOD	1	13.2±4.0	14.8±3.5$^{\# ns}$	15.0±3.7	13.1±3.9$^{\# ns}$	12.7±4.0	14.6±3.4$^{\# ns}$	11.7±3.2	15.4±3.3$^{\#<0.001}$
[NU/mL]	2	16.9±1.9	17.2±1.7$^{\# ns}$	17.7±2.0	16.5±1.6$^{\#<0.05}$	16.7±1.6	17.0±1.7$^{\# ns}$	16.6±1.7	17.2±1.5$^{\# ns}$
p		**<0.001**	**<0.01**	**<0.001**	**<0.001**	**<0.001**	**<0.01**	**<0.001**	**<0.05**
MnSOD	1	8.1±2.4	6.0±3.6$^{\#<0.01}$	7.5±3.6	7.3±2.7$^{\# ns}$	7.7±2.4	6.3±3.7$^{\# ns}$	7.57±2.49	6.52±3.39$^{\# ns}$
[NU/mL]	2	8.8±2.2	9.0±1.8$^{\# ns}$	9.4±2.4	8.6±1.7$^{\# ns}$	8.4±1.8	9.0±1.4$^{\# ns}$	8.35±1.77	8.97±1.43$^{\# ns}$
p		ns	**<0.01**	**<0.05**	**<0.05**	ns	**<0.05**	ns	**<0.01**
CuZnSOD	1	5.2±3.7	8.8±5.2$^{\#<0.01}$	7.4±4.6	5.8±4.7$^{\# ns}$	5.0±4.2	8.2±5.2$^{\#<0.05}$	4.11±4.0	8.86±4.35$^{\#<0.001}$
[NU/mL]	2	8.1±1.8	8.2±1.5$^{\# ns}$	8.3±1.8	8.0±1.6$^{\# ns}$	8.4±1.7	7.9±1.5$^{\# ns}$	8.20±1.94	8.27±1.16$^{\# ns}$
p		**<0.001**	ns	ns	**<0.05**	**<0.001**	ns	**<0.001**	ns
MDA	1	2.77±1.1	3.23±0.93$^{\# ns}$	2.8±0.72	2.9±1.2$^{\# ns}$	2.85±0.92	2.77±0.98$^{\# ns}$	2.67±0.94	3.02±0.90$^{\# ns}$
[umol/l]	2	2.20±0.7	2.31±0.83$^{\# ns}$	2.4±0.79	2.2±0.72$^{\# ns}$	2.12±0.65	2.33±0.91$^{\# ns}$	2.07±0.73	2.4±0.76$^{\# ns}$
p		**<0.01**	**<0.01**	**<0.05**	**<0.001**	**<0.001**	**<0.05**	**<0.01**	**<0.01**
LPS	1	604±341	606±287$^{\# ns}$	700±287	543±336$^{\# ns}$	541±319	620±320$^{\# ns}$	471±300	679±294$^{\#<0.01}$
[umol/l]	2	595±277	562±273$^{\# ns}$	686±275	514±258$^{\#<0.05}$	491±229	652±307$^{\#<0.05}$	465±212	667±284$^{\#<0.01}$
p		ns	ns	ns	ns	ns	ns	ns	ns
PSH	1	2.70±0.59	2.60±0.61$^{\# ns}$	2.77±0.52	2.63±0.61$^{\# ns}$	2.66±0.59	2.67±0.60$^{\# ns}$	2.65±0.59	2.67±0.57$^{\# ns}$
[umol/l]	2	3.00±0.74	2.85±0.42$^{\# ns}$	2.99±0.66	2.95±0.61$^{\# ns}$	2.89±0.62	3.08±0.68$^{\# ns}$	2.99±0.70	2.89±0.56$^{\# ns}$
p		**<0.01**	ns	ns	**<0.01**	ns	**<0.01**	**<0.01**	ns
CER	1	32.63±7.8	37.67±10.6$^{\#<0.05}$	35.3±10.1	33.7±8.7$^{\# ns}$	33.7±7.6	33.9±11.6$^{\# ns}$	32.1±7.3	35.8±10.5$^{\# ns}$
[mg/dl]	2	34.34±7.9	39.1±14.1$^{\# ns}$	37.1±13.1	35.6±9.0$^{\# ns}$	33.9±11.6	38.0±13.8$^{\# ns}$	34.1±9.0	37.4±12.7$^{\# ns}$
p		ns	ns	ns	ns	ns	**<0.01**	ns	ns
TAC	1	1.20±0.22	1.17±0.11$^{\# ns}$	1.23±0.22	1.17±0.17$^{\# ns}$	1.16±0.23	1.21±0.14$^{\# ns}$	1.14±0.24	1.22±0.13$^{\# ns}$
[mmol/l]	2	1.27±0.12	1.21±0.10$^{\# ns}$	1.29±0.11	1.21±0.10$^{\#<0.05}$	1.20±0.10	1.28±0.11$^{\#<0.01}$	1.20±0.09	1.28±0.11$^{\#<0.01}$
p		ns	ns	ns	ns	ns	**<0.01**	0.23	**<0.01**
TOS	1	5.52±4.80	6.72±4.38$^{\# ns}$	4.95±3.73	6.06±4.22$^{\# ns}$	5.73±4.02	5.33±3.57$^{\# ns}$	5.54±4.12	5.76±3.41$^{\# ns}$
[umol/l]	2	2.18±2.26	2.05±0.83$^{\# ns}$	2.00±1.24	2.18±2.23$^{\# ns}$	2.25±2.35	2.04±1.15$^{\# ns}$	2.41±2.43	1.88±1.14$^{\# ns}$
p		**<0.001**	**<0.001**	**<0.01**	**<0.001**	**<0.001**	**<0.01**	**<0.01**	**<0.001**
LPH	1	3.1±2.9	3.9±2.6$^{\# ns}$	3.06±2.50	3.33±2.55$^{\# ns}$	3.21±2.48	3.07±2.19$^{\# ns}$	2.99±2.50	3.43±2.13$^{\# ns}$
[umol/l]	2	1.2±1.5	1.2±0.6$^{\# ns}$	1.16±0.84	1.21±1.53$^{\# ns}$	1.25±1.56	1.13±0.92$^{\# ns}$	1.35±1.63	1.05±0.88$^{\# ns}$
p		**<0.001**	**<0.001**	**<0.01**	**<0.001**	**<0.01**	**<0.01**	**<0.01**	**<0.001**
OSI	1	5.18±4.39	5.83±3.92$^{\# ns}$	4.43±3.73	5.35±3.83$^{\# ns}$	5.31±4.0	4.48±3.1$^{\# ns}$	5.25±4.11	4.81±3.00$^{\# ns}$
	2	1.84±2.0	1.71±0.70$^{\# ns}$	1.67±0.98	1.85±1.99$^{\# ns}$	1.99±2.10	1.62±0.94$^{\# ns}$	2.06±2.17	1.57±0.88$^{\# ns}$
p		**<0.001**	**<0.001**	**<0.01**	**<0.001**	**<0.001**	**<0.01**	**<0.01**	**<0.001**

Legend: X_1 – before program; X_2 – after program; ns – not significant; SOD – superoxide dismutase; MnSOD - mitochondrial Mn-dependent superoxide dismutase; CuZnSOD – cytosolic Cu,Zn superoxide dismutase; MDA – malondialdehyde; LPS – lipofuscin; PSH – protein thiols; CER – ceruloplasmin; TAC – total non-enzymatic antioxidant capacity; TOS – total oxidant status; LPH – lipid peroxides; OSI – oxidative stress index.

Table S4. The differences between chosen parameters in normo- and hyperglycemia groups. Data are expressed as the mean ± SD, for comparison differences between measurements before the diet and after the diet within groups (p(1vs2)) and between groups (#p), a *t*-test was used. .

Parameter	X	Normoglycemia (*n* = 18)	Hyperglycemia (*n* = 35)	#*p*
Weight loss<br>[kg]		12.4 ±10.2	9.2±5.8	ns
VFA loss<br>[cm²]		21.1±18.4	21.2±21.6	ns
TBW loss<br>[kg]		2.69±3.1	2.26±3.91	ns
SMM loss<br>[kg]		2.11±2.67	2.07±3.70	ns
Glucose<br>[mg/dL]<br>p (1vs2)	1<br>2	89.8±7.8<br>81.5±14.2<br>ns	132.00±40.5<br>95.3±16.5<br>**<0.001**	**<0.001**<br>**<0.01**
FRU<br>[umol/L]<br>p (1vs2)	1<br>2	305.44±35.01<br>316.82±33.61<br>ns	365.01±84.62<br>313.49±49.43<br>**<0.01**	**<0.001**ns
FRU/PROT<br><br>p (1vs2)	1<br>2	4.05±0.42<br>4.32±0.64<br>ns	4.82±1.14<br>4.14±0.61<br>**<0.01**	**<0.01**ns
CRP<br>[mg/L]<br>p (1vs2)	1<br>2	4.00±5.83<br>3.64±3.48<br>ns	3.36±3.90<br>2.81±2.79<br>ns	ns<br>ns
SOD<br>[NU/mL]<br>p (1vs2)	1<br>2	11.9±3.3<br>16.9±1.5<br>**<0.001**	14.7±3.9<br>17.1±2.0<br>**<0.001**	**<0.01**<br>ns
MnSOD<br>[NU/mL]<br>p (1vs2)	1<br>2	6.6±3.0<br>8.4±2.1<br>ns	7.7±3.0<br>9.1±2.0<br>**<0.01**	ns<br>ns
CuZnSOD<br>[NU/mL]<br>p (1vs2)	1<br>2	5.3±4.5<br>8.5±2.1<br>**<0.05**	7.0±4.6<br>8.0±1.4<br>ns	ns<br>ns
MDA<br>[μmol/L]<br>p (1vs2)	1<br>2	2.7±1.1<br>2.1±0.8<br>**<0.05**	3.0±1.0<br>2.3±0.7<br>**<0.001**	ns<br>ns
LPS<br>[RF]<br>p (1vs2)	1<br>2	422.0±261.8<br>467.2±268.5<br>ns	692.8±312.3<br>639.0±261.2<br>ns	**<0.01**<br>**<0.05**
PSH<br>[μmol/L]<br>p (1vs2)	1<br>2	194.1±47.9<br>203.4±45.5<br>ns	205.8±44.8<br>230.0±49.5<br>**<0.01**	ns<br>ns
PSH/PROT<br><br>p (1vs2)	1<br>2	2.57±0.63<br>2.74±0.64<br>ns	2.71±0.57<br>3.04±0.63<br>**<0.01**	ns<br>ns
CER<br>[mg/dL]<br>p (1vs2)	1<br>2	35.5±10.3<br>39.0±14.9<br>ns	33.8±8.6<br>34.6±7.8<br>ns	ns<br>ns
TAC<br>[mmol/L]<br>p (1vs2)	1<br>2	1.12±0.17<br>1.19±0.09<br>ns	1.23±0.20<br>1.28±0.11<br>ns	ns **<0.01**
TOS	1	8.04±6.2	4.91±3.32	ns

[μmol/L]	2	2.57±0.66	1.92±2.20	ns
p (1vs2)		**<0.001**	**<0.001**	
LPH	1	4.50±3.71	2.86±2.06	ns
[μmol/L]	2	1.42±0.57	1.08±1.49	ns
p (1vs2)		**<0.001**	**<0.001**	

Legend: X_1 – before program; X_2 – after program; ns – not significant; VFA – visceral fat area; TBW – total body water; SMM – skeletal muscle mass; FRU – fructosamine; PROT – proteins; CRP – C-reactive protein; SOD – superoxide dismutase; MnSOD - mitochondrial Mn-dependent superoxide dismutase; CuZnSOD – cytosolic Cu,Zn superoxide dismutase; MDA – malondialdehyde; LPS – lipofuscin; PSH – protein thiols; CER – ceruloplasmin; TAC – total non-enzymatic antioxidant capacity; TOS – total oxidant status; LPH – lipid peroxides.
